# Literary Notes

**Published:** 1896-05

**Authors:** 


### Literary Notes.

The *Peoria Medical Record* is the name of a new journal that appeared February 15, 1896. It is a well-printed periodical and its arrangement is excellent. Dr. W. R. Allison is announced as the editor and Dr. C. H. Brobst is the managing editor.

The *Peoria Medical Journal*, that was published for some years and then discontinued, has been revived under its former editor, Dr. Thomas M. McIlvaine. The April issue presents a comely appearance and we wish it success.

The *Virginia Medical Semi-Monthly* has appeared in place of the *Virginia Medical Monthly*, and is a handsome quarto with double-column pages. Dr. Landon B. Edwards, the veteran editor and proprietor, continues at the helm, which is a guarantee of the success as well as excellence of the new journal.

---

THE following reports have been received: Eleventh annual report of the New York Post-Graduate Hospital; Twenty-fourth annual report of the Cincinnati Hospital; Report of the Dayton, O., Public Library and Museum; Annual report of the State Board of Charities; Statistics of the Trade and Commerce of Buffalo for the year 1895, issued by the Buffalo Merchants' Exchange. These are all interesting reviews of the work of these several institutions and corporations for the year.

---

MESSRS. REED & CARNRICK, of New York, have recently issued a brochure, entitled *Protonuclein Clinical Records, No. 2.* These records contain reports from leading physicians in hospitals and private practice, showing the favorable action of protonuclein in a large group of diseases. They also contain much valuable information as to indications for its use, dosage and methods. The pamphlet will be supplied on application to Messrs. Reed & Carnrick, 426-428 W. Broadway, New York.

---

WE ARE indebted to Messrs. Mariani & Co. for an interesting treatise entitled Coca and its Therapeutic Application. It is written by Monsieur Angelo Mariani, of Paris. It is beautifully illustrated, printed on handsome book paper, bound in dark blue muslin and contains the entire history of this valuable plant. It also treats of the physiology of coca and of its application in the treatment of the several diseases wherein its use is justified.

This admirable little book is published by Mr. J. N. Jaros, 52 W. Fifteenth street, New York, who will supply it gratuitously to physicians on application.

---

THE April *Monist* is replete with articles of philosophical, sociological and scientific interest. It opens with two articles on Röntgen's X rays, by leading European scientists. Edward Atkinson writes on the Philosophy of money; Professor Le Conte contributes From animal to man, and Professor Clark Murray on the Dualistic conception of nature. A number of other articles of interest are printed. It is published by the Open Court Publishing Co., Chicago, at $2.00 a year.

